# Supplementary material for: A fiber-pigtailed quantum dot device generating indistinguishable photons at GHz clock-rates
Source: Nanophotonics. 2025 Jan 6;14(11):1795–808. doi: 10.1515/nanoph-2024-0519 (PMC12133252; doi:10.1515/nanoph-2024-0519)
Supplement: Supplementary file 1 — Supplementary Material Details [file j_nanoph-2024-0519_suppl_001.pdf]

Supplementary Information for Manuscript A Fiber-pigtailed Quantum Dot Device Generating Indistinguishable Photons at GHz Clock-rates

Lucas Rickert[1,*], Kinga Żołnacz[2], Daniel A. Vajner[1], Martin von Helversen[1], Sven Rodt[1], Stephan Reitzenstein[1], Hanqing Liu[3,4], Shulun Li[3,4], Haiqiao Ni[3,4], Paweł Wyborski[5,6], Grzegorz Sęk[5], Anna Musiał[5], Zhichuan Niu[3,4,*], and Tobias Heindel[1,*]

[1] *Institute of Solid State Physics, Technical University Berlin, Hardenbergstraße 36, 10623 Berlin, Germany*
[2] *Department of Optics and Photonics, Wroclaw University of Science and Technology, Wybrzeże Stanisława Wyspiańskiego 27, 50-370 Wroclaw, Poland*
[3] *State Key Laboratory for Superlattice and Microstructures, Institute of Semiconductors, Chinese Academy of Sciences, Beijing 100083, China*
[4] *Center of Materials Science and Optoelectronics Engineering, University of Chinese Academy of Sciences, Beijing 100049, China*
[5] *Department of Experimental Physics, Wroclaw University of Science and Technology, Wybrzeże Stanisława Wyspiańskiego 27, 50-370 Wroclaw, Poland*
[6] *Department of Electrical and Photonics Engineering, Technical University of Denmark, 2800, Kgs. Lyngby, Denmark*
[*] *Corresponding author: lucas.rickert@tu-berlin.de, zcniu@semi.ac.cn, tobias.heindel@tu-berlin.de*

November 15, 2024

Supplementary Information

S1: FEM Simulations of FC-QD-hCBGs

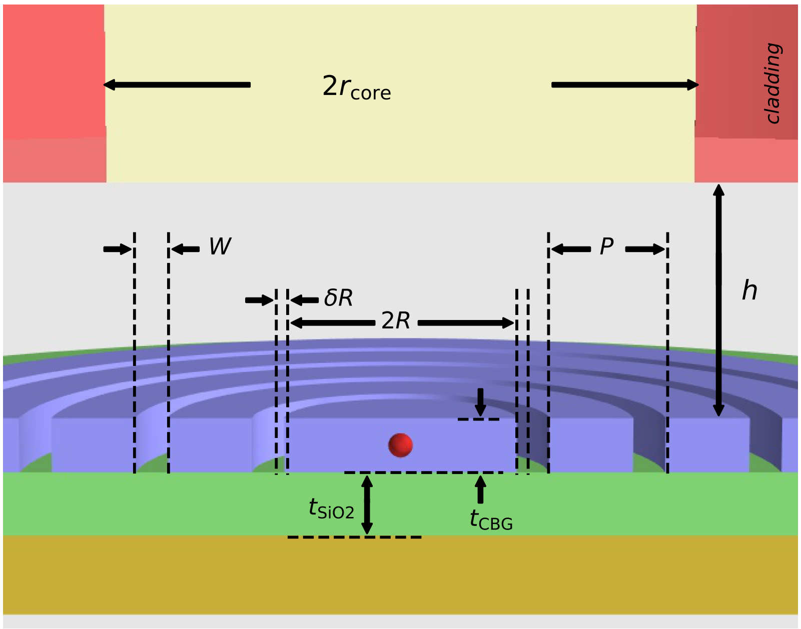

Fig. S1: (a) Schematic depiction of a QD-hCBG cavity with a UHNA3 fiber exhibiting core-radius r_{core} laterally aligned to the hCBG's center and separated by distance h. The hCBG cavity of thickness t_CBG consists of a central disc with radius $R + \delta R$ and a QD embedded in its center, surrounded by concentric rings of period P and grating gap width W. The hCBG cavity is situated on a SiO_2 layer of thickness t_{SiO_2} on gold.

R [nm]	δR	P [nm]	W [nm]	t_{CBG} [nm]	t_{SiO_2} [nm]	m_{Rings}	r_{core} [nm]
360	20	360	100	170	200	5	900

Tab. S1: hCBG cavity design parameters according to Fig. S1 and UHNA3 SMF dimensions. The parameter m_{Rings} represents the hCBG's number of rings.

n_{GaAs}	n_{SiO_2}	n_{Au}	n_{core}	n_{clad}
3.460	1.450	$0.12 + 6.33i$	1.493	1.451

Tab. S2: Refractive index parameters assumed in the FEM simulations for hCBG and UHNA3 SMF.

The FEM simulations presented in this work were performed using the commercial FEM software JCMSuite (JCMwave GmbH, 2024). The QD emitter is simulated by a TE dipole source situated in the centre of the central hCBG disc in both vertical and lateral direction. The in-fiber efficiency is calculated as the power obtained from the overlap of emitted CBG emission to the UHNA3-mode profile normalized to the total emitted dipole power. Further details on the simulation setup can be found in [1]. The detailed hCBG-cavity design parameters and used fiber-core radius are listed in Table S1, and the used refractive indices of the involved materials can be found in Table S2.

S2: Influence of fiber distance on simulated Purcell enhancement

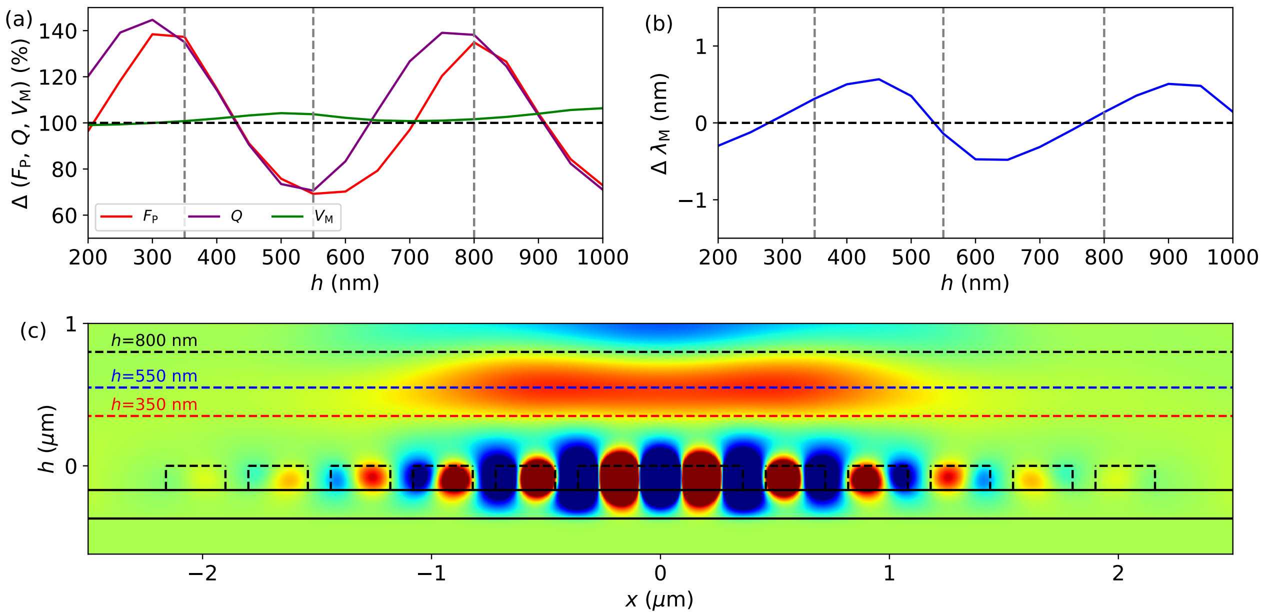

Fig. S2: (a) Simulated relative Purcell factor F_P, quality factor Q and mode volume V_M for varying fiber-to-hCBG distance h. The values are given in percent relative to the respective values without the present fiber, indicated as dashed black line. Q and V_M are obtained from eigenmode simulations, while F_P is obtained from scattering simulations at constant wavelength. (b) Simulated relative mode wavelength λ_P obtained from eigenmode simulations compared to the case without present fiber. (c) Field component E_X of the target hCBG mode without fiber present, obtained from eigenmode simulations.

As shown in Figure 1 in the main manuscript, the Purcell enhancement of a fiber-pigtailed QD-hCBG cavity depends noticeably on the fiber-to-cavity distance h. Figure S2 provides further insights on how the presence of the fiber in close proximity to the hCBG cavity influences the cavity parameters. Figure S2(a) shows the h-dependent simulated Purcell factor F_P, quality factor Q and mode volume V_M relative to the respective values of the hCBG cavity without the present fiber. It is apparent, that Q varies by up to 50% from the case without present fiber, while V_M stays nearly constant. It can therefore be assumed that the observed h dependency of F_P originates almost exclusively from $Q(h)$. Note that the displayed simulation results were obtained from eigenmode simulations of the target hCBG mode in the case of Q and V_M, while F_P results from scattering simulations at fixed wavelength which takes also contributions of other modes into account. This is the reason for the slight offset of Q and F_P. It is further found that the mode wavelength exhibits small shifts dependent on h, as displayed in Fig. S2.

The simulated $Q(h)$-dependency can be understood if the emitted field intensity of the hCBG cavity is considered. Fig. S2(c) shows the E_X field component of the hCBG mode, which shows a near-field pattern with nodes and anti-nodes in vertical direction. If the fiber is placed at the position of minimum field intensity, as is for $h = 350\,\text{nm}$, Q exhibits a maximum, while Q gets minimal if the fiber facet lies at positions of high field intensity, such as $h = 550\,\text{nm}$. The reason is that an abrupt change in refractive index, such as the transition of air to fiber-core, is detrimental to mode confinement, a concept that was for example used as "gentle-confinement-designs" for high Q photonic crystal cavities [2, 3].

We note that the vertical modulation of the hCBG mode's electric field is present without the fiber present. If a fiber is brought in close proximity in this way, the hCBG's mode confinement is altered by the induced effective refractive index changes for the given field distribution. The fiber adds additional vertical confinement, effectively becoming part of the hCBG cavity. The predicted noticeable influence of the hCBG mode's Q factor by the simulations renders this a versatile method to determine the fiber-to-cavity distance for fabricated structures in the experiment.

Note that Fig. S2(b) also predicts an h-dependent wavelength shift of the cavity mode of about $\pm 0.5\,\text{nm}$. The shifts between the cooldowns of the pigtailed QD-hCBG device shown in Fig. 2(c) are however larger than expected from the simulations, indicating that the built-up strain might cause a change of the refractive index as mentioned in the main manuscript.

S3: Experimental setup

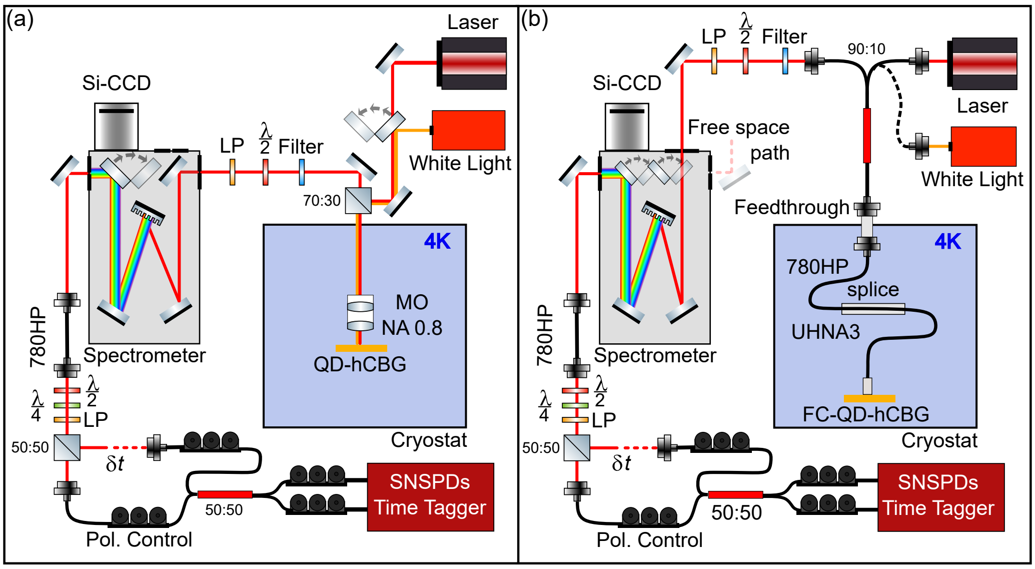

Fig. S3: (a) Schematic depiction of the experimental setup used for free-space characterization of the hCBG cavity before the fiber pigtailing and (b) for the FC-device after the fiber-pigtailing. MO: microscope objective. LP: linear polarizer

Figure S3 shows schematics of the experimental setup used for the quantum-optical measurements of the sample before and after the fiber-pigtailing. For the measurements before the pigtailing in the setup configuration in (a), the QD-hCBG cavity sample is placed in a closed-cycle helium cryostat and cooled to 4 K with a low-temperature compatible microscope objective with NA=0.8 for confocal excitation/emission experiments. Excitation light reaching the sample via the excitation path is either from a pulsed 80 MHz laser system with nominal 2 ps temporal pulse-width (picoEmerald, APE GmbH) or from a broadband white-light source (SLS201L/M, Thorlabs GmbH). The emission from the sample exits the cryostat towards a 0.75 m spectrograph (entrance slit width: 100 μm) with 1200 g/mm grating, using a half-wave-plate before the monochromator to match the polarization to the grating. For p-shell experiments, the emission from the sample is filtered with a bandpass filter ($\lambda_{\text{center}} = (940 \pm 5)$ nm) before entering the monochromator. To spectrally resolve the signal, the emission is reflected from the grating onto a 1340 pixel Si-CCD camera cooled to 200 K with a Peltier element. For time-resolved measurements, the emission is reflected from the grating trough a separate spectrograph's exit (exit slit width: 80 μm) into a single mode fiber (SMF 780HP). We expect the spectral filtering caused by the grating and SMF in-coupling to be around 100 μeV (0.07 nm). The SMF leads to a Hong-Ou-Mandel setup set as follows: An initial free-space polarization control consisting of half-waveplate, quarter-waveplate and linear polarizer ensures equal splitting at a polarizing BS to match both arms of the Mach-Zehnder interferometer (MZI) in intensity. The arms of the MZI are precisely time-matched for the time-difference δt ($\delta t = 2.0$ ns and 12.5 ns for 80 MHz excitation rate, and $\delta t = 781$ ps for 1.28 GHz excitation rate). The interference then occurs in a 50:50 fiber-beamsplitter, where the input polarizations are fully-controlled by polarization paddles in fiber to set the co- and cross-polarized interference. The output of the 50:50 fiber-beamsplitter leads to superconducting nanowire single photon detectors (SNSPDs) (Single Quantum EOS CS, Single Quantum B.V.) connected to time tagging electronics (QuTag, qtools GmbH). Additional polarization paddles on the fibers leading to the detector match the polarization to the nanowire orientation. The correct setting of polarization and time delay in the MZI is confirmed by interfering laser pulses at the QD emission wavelength and observing close to 100% interference contrast.

For the measurements of the FC-device, the sample was placed in the cryostat and the 780HP fiber output of the UHNA3-780HP fiber patchcord was connected to the cryostats vacuum fiber-feedtrough. On the room temperature side of the feedtrough, a 90:10 fiber-based beamsplitter was connected, whose 10% input arm was used to connect the above mentioned 80 MHz pulsed laser for optical excitation, or the broadband white-light source for reflection measurements. The 90% fiber-beamsplitter output was connected to a free-space out-coupler towards the spectrometer. The same spectrometer and Si-CCD was used as for the free-space measurements, but was entered from an alternative entrance.

The efficiency estimation for the FC setup is discussed in S.I., section S8.

S4: Fiber-pigtailed spectra during cooldown

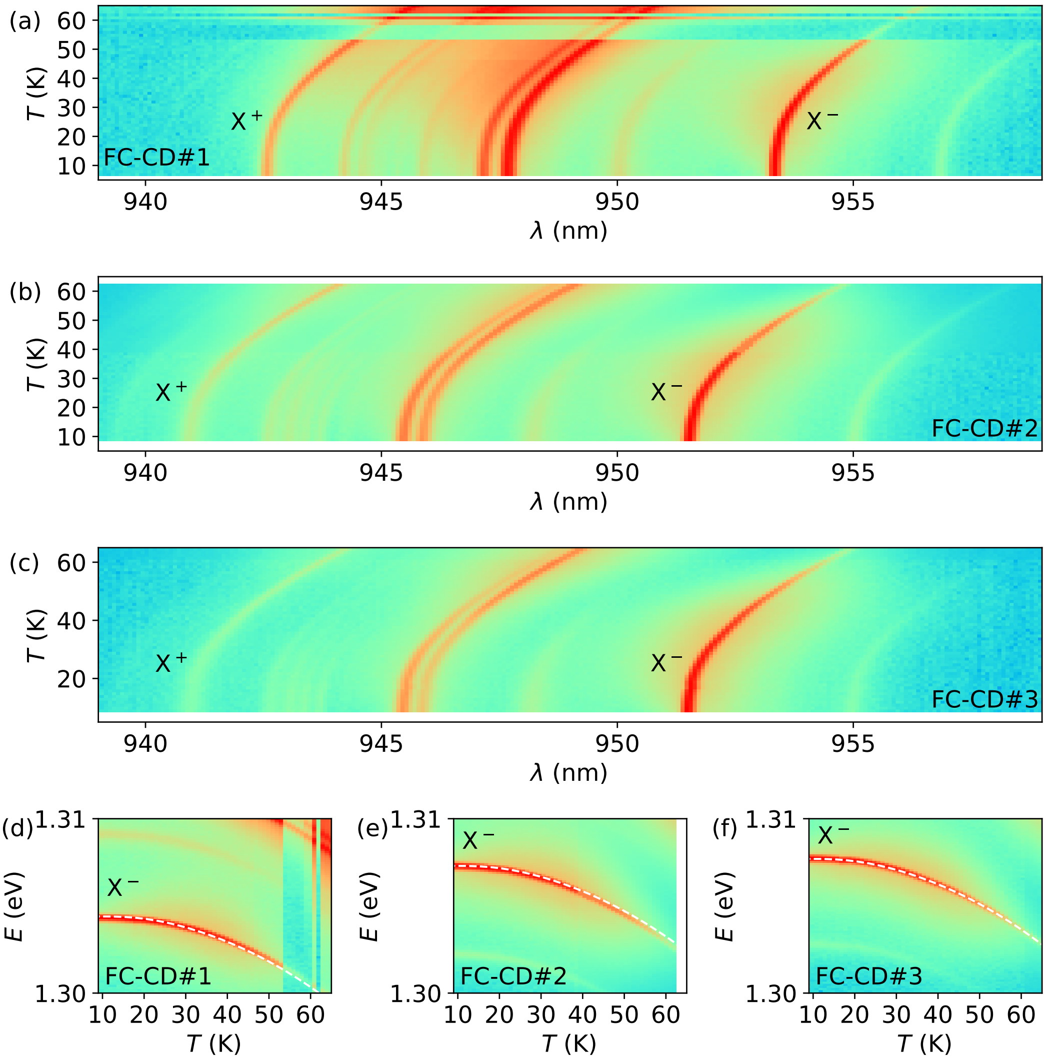

Fig. S4: Recorded PL-spectra under above-band excitation during the cooldowns (a) #1, (b) #2 and (c) #3 in logarithmic scale. The intensity variations in (a) at temperatures >54 K stem from changes in the excitation power to check for saturation. (d)-(f) show the X^- emission, respectively, with a dashed line following equation (1).

Figure S4 shows the temperature dependent spectra during the three cooldowns of the FC-device for an excitation wavelength of $\lambda_{exc} = 793\,nm$. For cooldown #1, the excitation power conditions at the QD were unknown, and the excitation power and integration time on the CCD was adjusted in the range above 54 K to check for QD state saturation. The changes in intensity above 54 K stem from these excitation power variations, and not from the sample itself. Once the cooldown was started, it could not be stopped until base temperature was reached, causing the intensity fluctuations to be recorded.

Fig. S4(d)-(f) show the temperature-dependent emission of the X^- line as a close-up for the respective cooldowns. The dotted line follows equation (1) for a temperature dependent band gap energy E_g of GaAs (shifted by a set confinement energy for each cooldown), with the corresponding parameters listed in [4]:

$$E_g = E_0 - \alpha \left(\frac{W_1 \Theta_1}{\exp(\Theta_1/T) - 1} + \frac{W_2 \Theta_2}{\exp(\Theta_2/T) - 1} \right). \quad (1)$$

The good agreement with theory indicates that the FC-device reaches the indicated temperatures (ultimately 4.8 K), and the wavelength shifts compared to the sample before pigtailing cannot be explained by temperature, as elaborated in the main text. We note further that equation (1) with InAs parameters does not fit the experimental data well, indicating that the QD's temperature dependence is either largely determined by the surrounding GaAs matrix material, or significant Ga incorporation takes place during the growth.

S5: Assigment of QD states to emission lines.

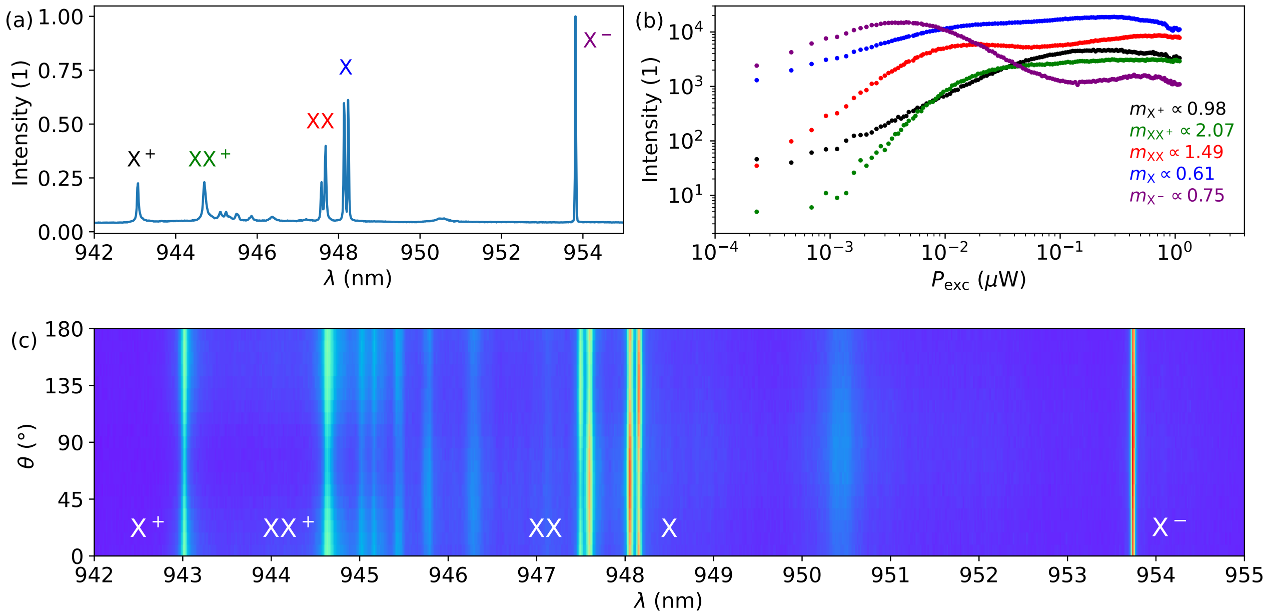

Fig. S5: **Excitation power series and polarization series measurements to identify the QD states of the fiber-pigtailed QD-hCBG device**. The measurements were obtaiend for Cooldown #1 under off-resonant excitation with $\lambda_{exc} = 793\,\text{nm}$ at $T = 4.8\,\text{K}$. (a) Spectrum of the fiber-pigtailed QD-hCBG device with assigned QD-states. (b) Peak intensity of the labeled states in (a) for varying excitation powers P_{exc}. The slope m obtained from linear fits of the data in this double-logarithmic scale is listed. (c) Polarization resolved spectra under off-resonant excitation for detection angles θ with indicated QD states.

To assign the observed emission lines in the spectrum of the fiber-pigtailed QD-hCBG device excitation power dependent and detection polarization dependent measurements were conducted during during the first cooldown. The data is displayed in Figure S5, with the assigned states for the spectrum shown in Fig. S5(a).

The XX and X emission lines are assigned by the visible fine-structure splitting in the polarization resolved detected emission intensity in Fig. S5(c), and X is distinguished from XX based on the lower slope from the excitation power dependent measurements in Fig. S5(b).

Neither of the assigned X$^+$, XX$^+$ and X$^-$ states show a (resolvable) fine-structure splitting for varying polarization, indicating trionic states. X$^+$ and XX$^+$ are distinguished based on their power dependency. The appearance of X$^+$ and XX$^+$ at shorter wavelength (i.e. anti-binding) compared to the X is commonly observed for InAs QDs, while the negative trion appears at longer wavelengths (i.e. binding) compared to X [5].

S6: Off-resonant spectra before and after fiber-pigtailing

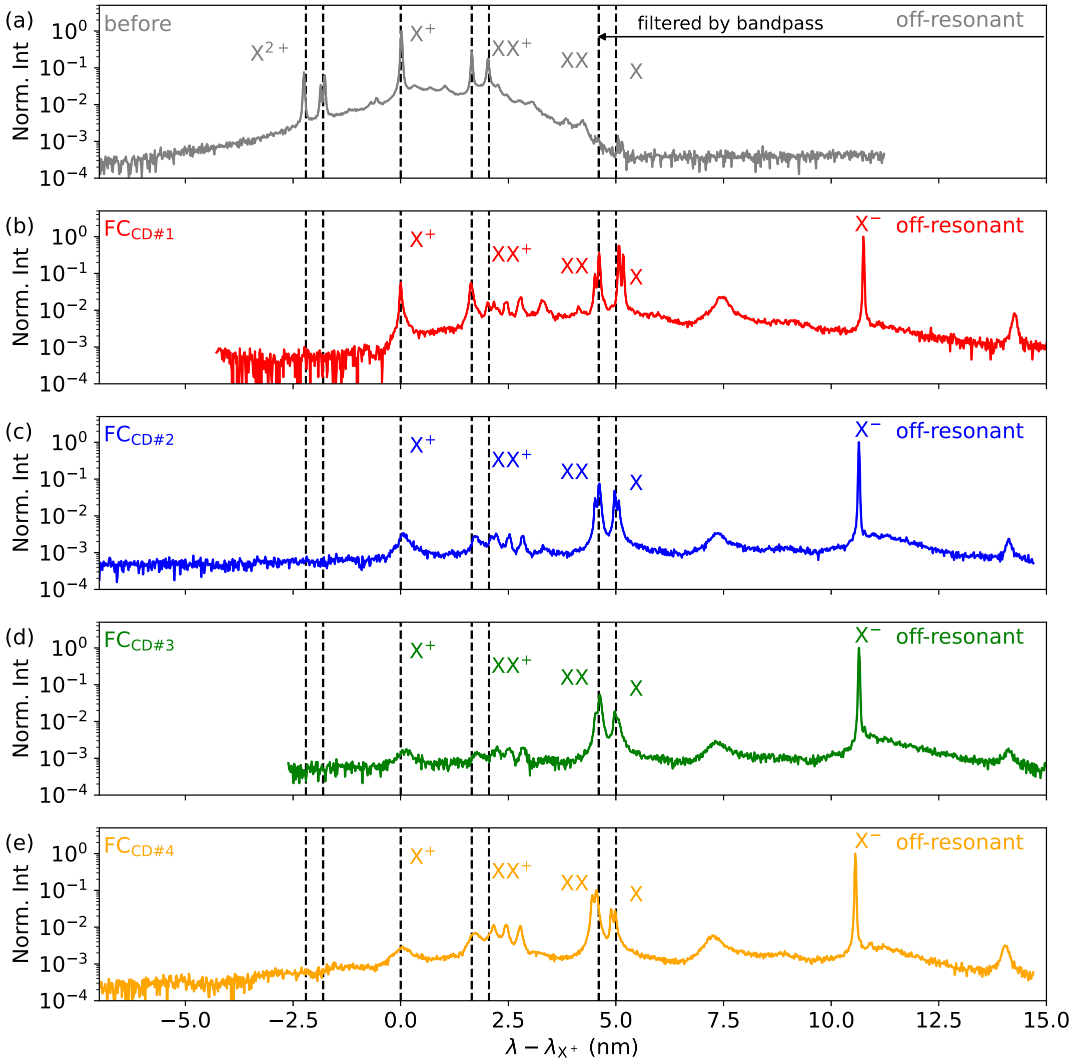

Fig. S6: PL-spectra under above-band ($\lambda_{\text{exc}} = 793\,\text{nm}$) excitation (a) before and (b-e) after the fiber-pigtailing for the respective cooldowns. The normalized intensity is displayed in logarithmic scale. The recorded emission wavelength is normalized to the emission wavelength of the X^+. The spectral positions of associated QD states are indicated as dashed lines. For the measurement before the pigtailing, the filtered-out spectral region caused by the bandpass filter is indicated.

Figure S6 shows the spectra in Fig. 2(a) in the main text under off-resonant excitation ($\lambda_{\text{exc}} = 793\,\text{nm}$) before and after the fiber-pigtailing plotted in logarithmic scale. The wavelength on the x-axis is normalized to the emission wavlength of the X^+ transition. Respective QD states corresponding to emission lines are indicated, as well as the filtered-out spectral region by the employed bandpass filter for the spectrum before the coupling.

X^+, XX+, XX and X lines appear at identical spectral positions, clearly proving that the fiber-pigtailing was deterministic for the pre-selected QD-hCBG cavity. The strong emission background in the spectrum before the pigtailing in Fig. S6(a) is caused by $\sim 10\times$ higher excitation power P_{exc}. Similarly, P_{exc} was slightly higher for cooldowns #1 and #4 for the displayed spectra, causing slightly varying backgrounds. An excition wavlength-dependent comparison before and after the pigtailing is discussed further in S.I., section S7.

S7: Photoluminescence excitation before and after fiber-pigtailing

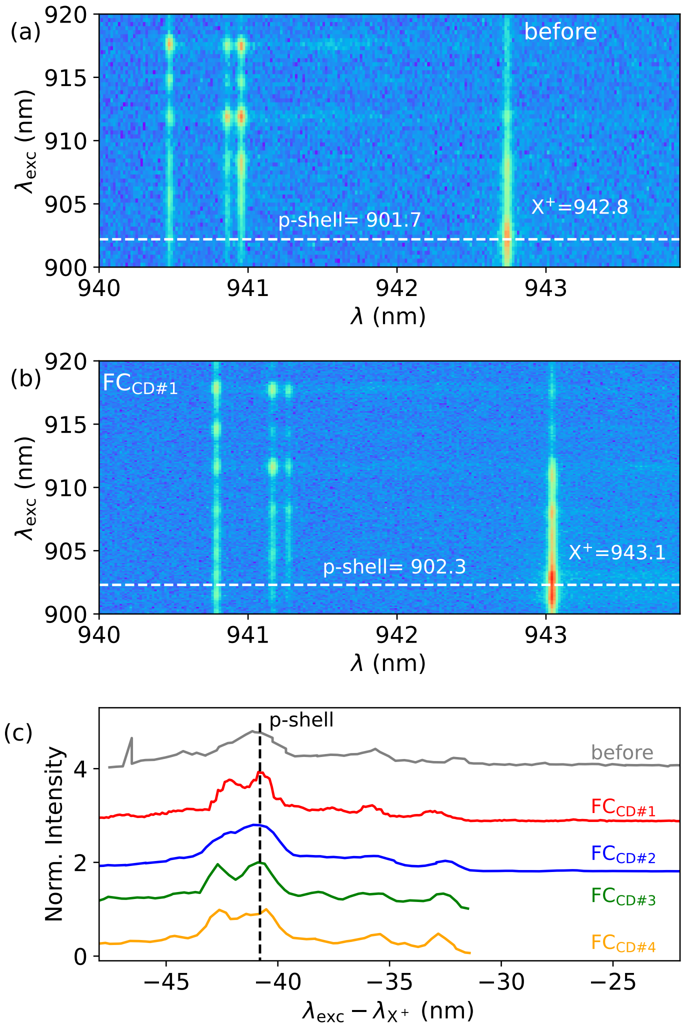

Fig. S7: Photoluminescence excitation (PLE) scans for varying excitation wavelengths λ_{exc} and corresponding emission wavelength λ. (a) PLE before and (b) after the fiber-pigtailing for cooldown #1. The respective p-shell is indicated. (c) Intensity of the X^+ emission line for PLE scans before and after the pigtailing for respective cooldowns with indicated p-shells.

Figure S7 shows photoluminescence excitation (PLE) data before and after the pigtailing, with detailed PLE scans before in Fig. S7(a) and after the pigtailing for cooldown #1 in in Fig. S7(b). The respective p-shell and X^+ wavelengths are indicated. Fig. S7(c) shows the intensity of the X^+ line for varying excitation wavelengths normalized to the trion emission wavelengths, i.e. the s-p splitting. The fact that identical s-p splitting is observed before and after the pigtailing proves the deterministic fiber-pigtailing technique.

S8: Setup efficiency estimation

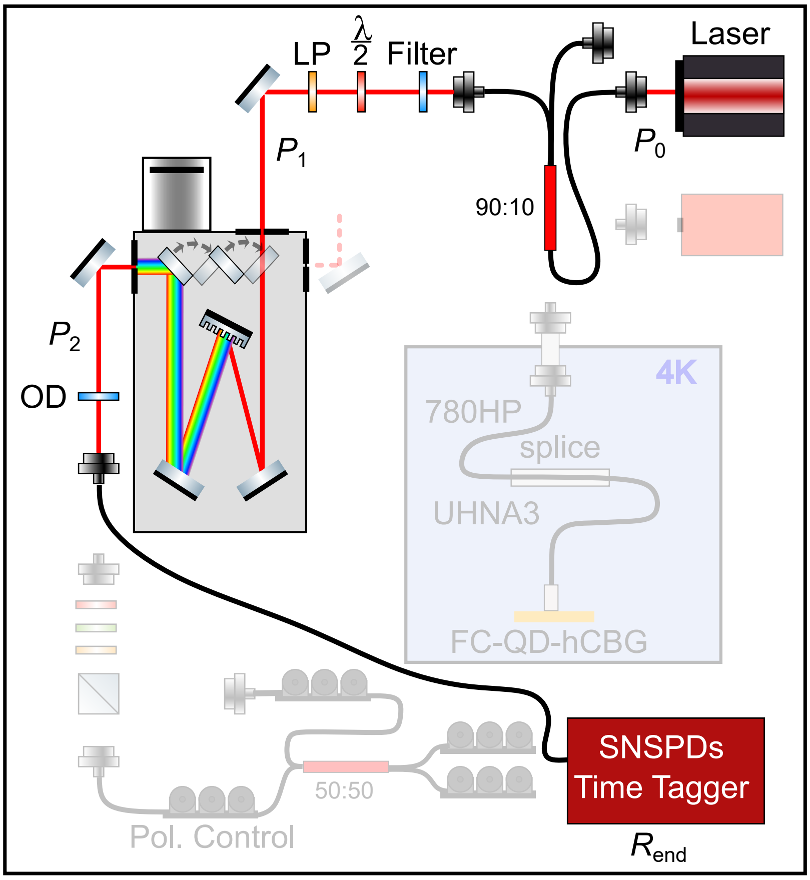

Fig. S8: Schematic depiction of the measurement to estimate the efficiency of the setup for the FC-device.

Figure S8 shows the measurement to estimate the setup efficiency for the characterisation of the fiber-pigtailed sample. The fiber-based beamsplitter's input, which would be connected to the cryostat's fiber-feedthrough for the characterization of the FC-device, is connected to a continuous-wave (cw) laser at λ_{X+} with input power $P_0 = 5\,\mu$W. The 10% output of the fiber-based beamsplitter is blocked, and the cw-laser signal reaches the spectrometer via the 90% output. The power P_1 is measured at the entrance of the spectrometer. The power P_2 is the cw-laser power at the exit of the spectrometer, and is attenuated with OD-filters before entering the fiber towards the SNSPD detectors. R_{end} is the observed countrate on the SNSPDs.

We find $P_1/P_0 = 0.327$, $P_2/P_1 = 0.333$ and $(R_{\mathrm{end}}hc/\lambda_{X+})/P_2 = 0.45$. This leads to an overall setup efficiency from the fiber-based beamsplitter input to the detectors of 0.049. For the experimental in-fiber efficiency at the UHNA3 fiber-facet, the efficiency by the splice (0.95) and by the vacuum fiber-feedthrough (0.6) have to be additionally taken into account, as described in the main text.

S9: Influence of T_1 on V_{HOM}

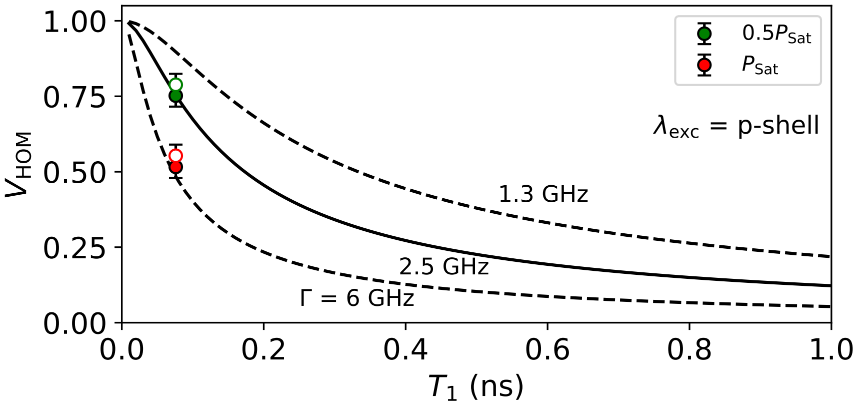

Fig. S9: Measured HOM visibilities V_{HOM} at $\delta t = 12.5\,\mathrm{ns}$ for observed T_1-time of the fiber-pigtailed device and varying fraction of the saturation power P_{sat}. The indicated lines correspond to equation (2) for different Gaussian line-broadening Γ.

The following S.I. provides a reference for the measured two-photon-indistinguishablity V_{HOM} values in p-shell excitation for the fiber-pigtailed device, since we did not measure V_{HOM} before the pigtailing. The T_1-influence on the indistinguishability of two emitted photons can be described as [6, 7]:

$$V_{\mathrm{HOM}}(T_1, \Gamma) = \frac{A(\Gamma)}{T_1} \exp\left(\frac{A(\Gamma)^2}{\pi T_1^2}\right) \cdot \mathrm{erfc}\left(\frac{A(\Gamma)}{\sqrt{\pi} T_1}\right) \tag{2}$$

with the complementary error-function erfc(), factor $A(\Gamma) = \frac{\sqrt{\ln 2}}{\sqrt{2}\pi\Gamma}$, and the inhomogeneously broadened QD emission line following a Gaussian distribution with FWHM-linewidth Γ. Figure S9 shows the measured V_{HOM} values of the pigtailed QD-hCBG device for p-shell excitation at different fractions of P_{sat} discussed in the main text plotted against the observed T_1-time. The results of equation (2) are shown for Γ-values between 1.3 GHz and 6 GHz (i.e., linewidths of 0.005 nm and 0.028 nm, respectively), in agreement with inhomogenous-broadening values previously reported for InAs QD-hCBG cavities under p-shell excitation [8]. The photon-indistinguishability observed for the fiber-pigtailed device, as discussed in the main text, are hence very similar to free-space operated QD-hCBG cavities. It can be expected, that even higher indistinguishabilities under non-resonant excitation can be reached, if T_1 can be further reduced.

S10: Additional data from a second fiber-pigtailed QD-hCBG device

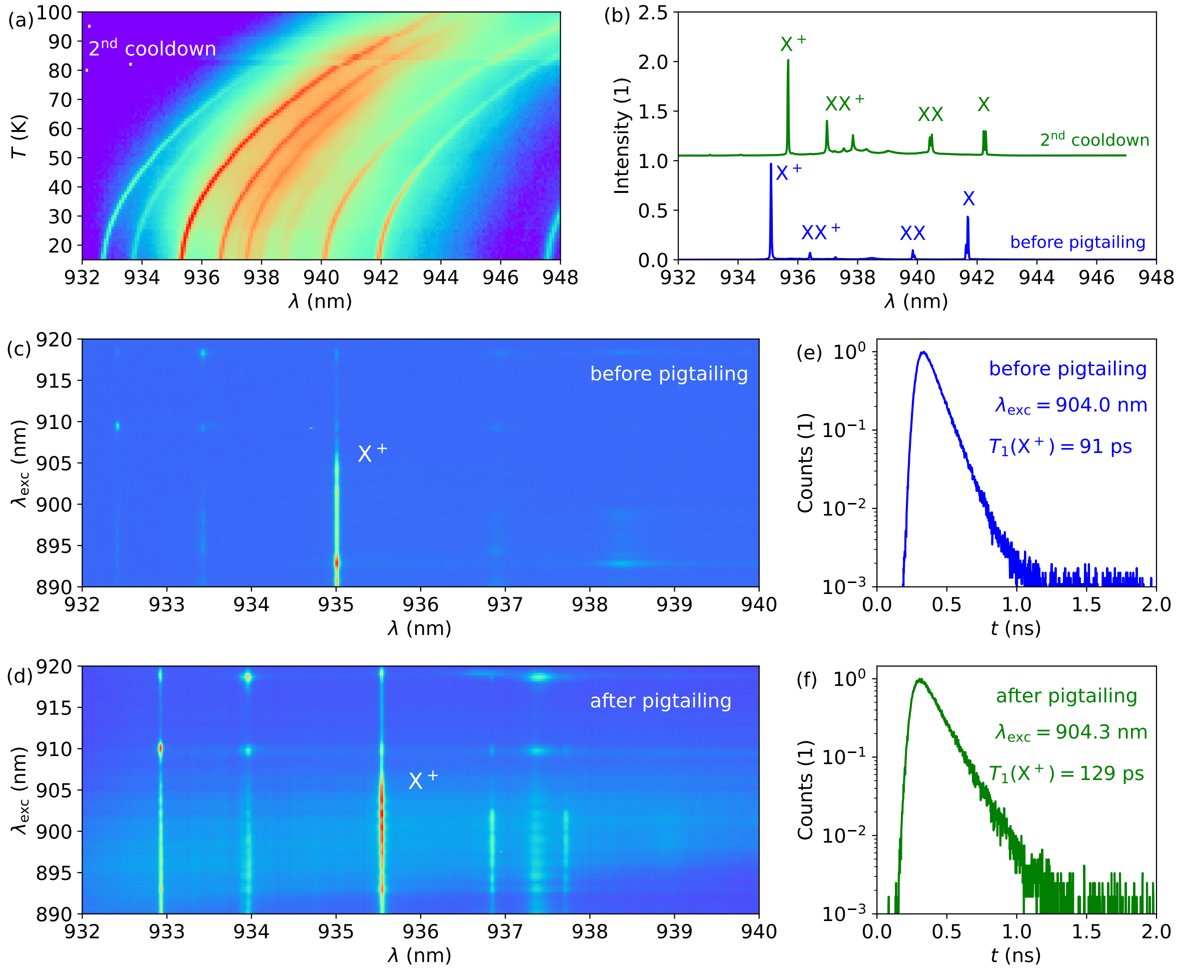

Fig. S10: **Additional data for the second fiber pigtailed QD-hCBG device FC-CBG #2.** (a) Emission spectra under $\lambda_{\text{exc}} = 793\,\text{nm}$ excitation during the second cooldown after the pigtailing. (b) Emission spectra at base temperature before and after the pigtailing. (c) PLE spectra before and (d) after the pigtailing. (e) T_1-time of X^+ for p-shell excitation before and (f) after the fiber-pigtailing.

We performed a fiber-pigtailing process for a second sample. In the following, we will refer to this second pigtailed as FC-CBG #2. In contrast to the discussed pigtailed device in the main manuscript, device FC-CBG #2 showed a sudden decrease in emission intensity a couple of minutes after reaching base temperature in a first cooldown. The emission properties observed during a second cooldown are displayed in Figure S10.

Figure S10(a) shows recorded spectra under off-resonant excitation during the second cooldown. Figure S10(b) shows the device's emission at $T = 4.8\,\text{K}$ in green, as well as the spectrum before the pigtailing in blue. Despite the reduced intensity after the first cooldown, the fiber-to-cavity alignment was not completely lost, and further spectra, photoluminescence excitation (PLE) scans and time-resolved measurements could be conducted during the second cooldown, as displayed in Figure S10(c-f). Based on the observed quasi-resonances in the PLE and the spectral positions of the assigned QD states, the pigtailed QD-hCBG device is unambiguously identified as the target device before the pigtailing, albeit again with a slight red shift. The measured T_1-time of X^+ appears to be once again slower than before the pigtailing. Note that for this device FC-CBG #2, it was not possible to obtain information on the cavity mode from reflection measurements, since the signal-to-noise ratio was too low stemming from the fiber-displacement, so no information can be gained on the mode's Q-factor after the pigtailing.

To put this observed properties of device FC-CBG #2 in perspective with the pigtailed device in the main manuscript: we believe that the pigtailed device discussed in the manuscript experienced an elastic deformation and strain built-up during the first thermal cycle, which appears to equilibrated after the first cooldown. For device FC-CBG #2, the thermal stressed appeared to be enough to cause a plastic deformation of the fiber-to-hCBG connection, which resulted in the observed deteriorated alignment and loss in emission intensity. The plastic deformation however, is also believed to have caused a partial relaxation of the strain accumulated during

the first cooldown. This can explain why there are no intensity shifts observed in this second sample, compared to the device discussed in the manuscript.

The reason why device FC-CBG #2 showed a plastic deformation, while the device discussed in the manuscript had intact fiber-alignment after the first cooldown could originate from several factors: Firstly, the temperature ramp of the cryostat during 1st cooldown of device FC-CBG #2 was steeper than for the second cryostat used for the later cooldowns, potentially causing higher thermal stress. Secondly, we think that the membrane area being in contact with the fiber might play a crucial part in the strain distribution and potential deformations.

Supplementary Information References

[1] L. Rickert, T. Kupko, S. Rodt, S. Reitzenstein, and T. Heindel, "Optimized designs for telecom-wavelength quantum light sources based on hybrid circular Bragg gratings," *Optics Express*, vol. 27, p. 36824, Dec. 2019.

[2] Y. Akahane, T. Asano, B.-S. Song, and S. Noda, "High-Q photonic nanocavity in a two-dimensional photonic crystal," *Nature*, vol. 425, pp. 944–947, Oct. 2003.

[3] Y. Tanaka, T. Asano, and S. Noda, "Design of Photonic Crystal Nanocavity With Q-Factor of $\sim 10^9$," *Journal of Lightwave Technology*, vol. 26, pp. 1532–1539, June 2008.

[4] R. Pässler, "Temperature dependence of fundamental band gaps in group IV, III–V, and II–VI materials via a two-oscillator model," *Journal of Applied Physics*, vol. 89, pp. 6235–6240, June 2001.

[5] S. Rodt, A. Schliwa, K. Pötschke, F. Guffarth, and D. Bimberg, "Correlation of structural and few-particle properties of self-organized In As / Ga As quantum dots," *Physical Review B*, vol. 71, p. 155325, Apr. 2005.

[6] P. Gold, A. Thoma, S. Maier, S. Reitzenstein, C. Schneider, S. Höfling, and M. Kamp, "Two-photon interference from remote quantum dots with inhomogeneously broadened linewidths," *Physical Review B*, vol. 89, p. 035313, Jan. 2014.

[7] C. Nawrath, H. Vural, J. Fischer, R. Schaber, S. L. Portalupi, M. Jetter, and P. Michler, "Resonance fluorescence of single In(Ga)As quantum dots emitting in the telecom C-band," *Applied Physics Letters*, vol. 118, p. 244002, June 2021.

[8] L. Rickert, D. A. Vajner, M. von Helversen, J. Schall, S. Rodt, S. Reitzenstein, H. Liu, S. Li, H. Ni, Z. Niu, and T. Heindel, "High Purcell-enhancement in quantum-dot hybrid circular Bragg grating cavities for GHz-clockrate generation of indistinguishable photons," Aug. 2024. arXiv:2408.02543 [quant-ph].
